# Complete genome sequence of *Candidatus* Ruthia magnifica

**DOI:** 10.4056/sigs.1103048

**Published:** 2010-10-27

**Authors:** Guus Roeselers, Irene L. G. Newton, Tanja Woyke, Thomas A. Auchtung, Geoffrey F. Dilly, Rachel J. Dutton, Meredith C. Fisher, Kristina M. Fontanez, Evan Lau, Frank J. Stewart, Paul M. Richardson, Kerrie W. Barry, Elizabeth Saunders, John C. Detter, Dongying Wu, Jonathan A. Eisen, Colleen M. Cavanaugh

**Affiliations:** 1 Harvard University, Department of Organismic and Evolutionary Biology, 16 Divinity Avenue, Biolabs 4080, Cambridge, MA 02138, USA.; 2 Radboud University, Department of Microbiology, Heyendaalseweg 135, 6525 AJ Nijmegen, The Netherlands.; 3 Department of Biological Sciences, 106 Central St, Wellesley, MA 02482, USA.; 4 Department of Energy Joint Genome Institute, 2800 Mitchell Drive, Walnut Creek, CA 94598, USA.; 5 Harvard Medical School, Department of Microbiology and Molecular Genetics, 200 Longwood Avenue, Boston, MA 02115, USA.; 6 University of California, Davis Genome Center, Genome and Biomedical Sciences Facility, Room 5311, 451 East Health Sciences Drive, Davis, CA 95616–8816, USA.

**Keywords:** HYDROTHERMAL VENT, CLAM, SULFUR, SYMBIOSIS, CHEMOSYNTHESIS, VESICOMYIDAE

## Abstract

The hydrothermal vent clam *Calyptogena magnifica* (*Bivalvia*: *Mollusca*) is a member of the *Vesicomyidae*. Species within this family form symbioses with chemosynthetic *Gammaproteobacteria*. They exist in environments such as hydrothermal vents and cold seeps and have a rudimentary gut and feeding groove, indicating a large dependence on their endosymbionts for nutrition. The *C. magnifica* symbiont, *Candidatus* Ruthia magnifica, was the first intracellular sulfur-oxidizing endosymbiont to have its genome sequenced (Newton *et al*. 2007). Here we expand upon the original report and provide additional details complying with the emerging MIGS/MIMS standards. The complete genome exposed the genetic blueprint of the metabolic capabilities of the symbiont. Genes which were predicted to encode the proteins required for all the metabolic pathways typical of free-living chemoautotrophs were detected in the symbiont genome. These include major pathways including carbon fixation, sulfur oxidation, nitrogen assimilation, as well as amino acid and cofactor/vitamin biosynthesis. This genome sequence is invaluable in the study of these enigmatic associations and provides insights into the origin and evolution of autotrophic endosymbiosis.

## Introduction

Chemosynthetic symbioses, initially discovered at hydrothermal vents, also exist in shallow mud flats and seagrass beds, and deep sea cold methane seeps [1]. In each case it is clear that these symbioses play major roles in community structuring and sulfur and carbon cycling. However, despite the widespread occurrence of these partnerships, little is known of the intricacies of host-symbiont interaction or symbiont metabolism due to their inaccessibility and our inability to culture either partner separately.

The giant clam, *Calyptogena magnifica* Boss and Turner (Bivalvia: Vesicomyidae), was one of the first organisms described after the discovery of hydrothermal vents. Vesicomyidae is a relatively old family, with fossil records and phylogenies dating them at 50-100 Ma [2]. *C. magnifica* grows to a large size (>26 cm in length), despite having a reduced gut and ciliary food groove [3], presenting a conundrum regarding how it acquires sufficient nutrients. The discovery of chemoautotrophic, Gammaproteobacterial endosymbionts, now named *Candidatus* Ruthia magnifica (in memory of Prof. Ruth Turner), within *C. magnifica* gill bacteriocytes [4,5] helped to solve the mystery surrounding the nutrition of this clam. The host depends largely on these endosymbionts for its carbon, as indicated by its anatomy and by stable carbon isotopic ratios [6]. However, how the host satisfies the rest of its nutritional requirements remained unknown.

Vesicomyid symbionts are presumed to be obligately symbiotic as they have a relatively reduced genome size [7-9], and are transmitted vertically between successive host generations via the egg [10]. Evidence has been presented indicating a single Gammaproteobacterial symbiont is present in vesicomyids that have been examined via rRNA phylotyping [11]. However recent evidence suggests that vesicomyids may harbor two symbiont phylotypes, both of which fall into the same clade but are distinct phylotypes. Thus the clams may acquire divergent symbionts laterally via uptake from an environmental population or horizontal transfer from co-occurring hosts [12].

**Fig 1:**
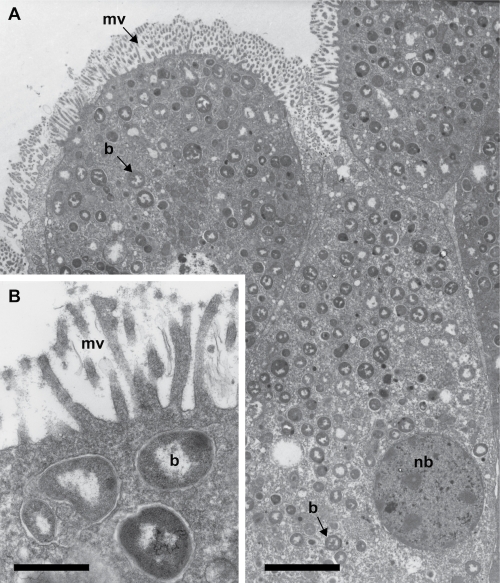
Transmission electron micrographs of *Candidatus* R. magnifica within host bacteriocytes. (A) Bacteriocyte containing many small (0.3 μm) coccoid-shaped symbionts. Scale bar = 5 μm. (B) Higher magnification. Scale bar = 0.4 μm. mv = microvilli, nb = bacteriocyte nucleus, b = *Candidatus* R. magnifica. (figure adapted from Cavanaugh [1983]).

**Fig 2:**
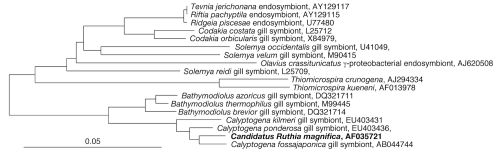
Phylogenetic tree inferred from complete 16S rRNA gene sequences of *Candidatus* R. magnifica, several chemoautotrophic symbionts of marine invertebrates, and two ‘freeliving’ *Thiomicrospira* species. The tree was calculated using the Neighbor-Joining algorithm with Kimura 2-parameter correction. The tree was rooted with *Fusobacterium perfoetens* (M58684), which was pruned from the tree.

Here we present a classification and a set of features (Figure 1, Figure 2, Table 1) for *Candidatus* R. magnifica, together with a description of the complete genome sequence and annotation originally presented in [9].

**Table 1:** Classification and general features of *Candidatus* Ruthia magnifica according to the MIGS recommendations.

**MIGS ID**	**Property**	**Term**	**Evidence code**^a^
MIGS-2	Current classification	Domain *Bacteria* Phylum *Proteobacteria* Class *Gammaproteobacteria* Gammaproteobacteria unclassified sulfur-oxidizing symbionts Candidatus *Ruthia magnifica*	TAS[9]
	Gram stain	negative	NAS
	Cell shape	coccus	TAS[4,5]
	Motility	none	TAS[4,5]
	Sporulation	nonsporulating	NAS
	Temperature range	mesophile	NAS
	Optimum temperature	unknown	
	Carbon source	CO_2_	TAS[6]
	Energy source	H_2_S (Chemoautotroph)	TAS[6]
	Terminal electron receptor	O_2_	TAS[6]
MIGS-6	Habitat	endosymbiont, marine, host, hydrothermal vents	TAS[3,4,5]
MIGS-6.3	Salinity	~34.6 pps	NAS
MIGS-22	Oxygen	aerobic	TAS [4,9]
MIGS-15	Biotic relationship	symbiotic	TAS [4,9]
MIGS-14	Pathogenicity	none	NAS
MIGS-4	Geographic location	9-North, East Pacific Rise, Hydrothermal vents	
MIGS-5	Sample collection time	December 2004	TAS [9]
MIGS-4.1 MIGS-4.2	Latitude Longitude	9° 51’ N 104° 18’ W	NAS
MIGS-4.3	Depth	~2500 m	NAS

**a)** Evidence codes - IDA: Inferred from Direct Assay; TAS: Traceable Author Statement (i.e., a direct report exists in the literature); NAS: Non-traceable Author Statement (i.e., not directly observed for the living, isolated sample, but based on a generally accepted property for the species, or anecdotal evidence). These evidence codes are from the Gene Ontology project [13].

## Organism information

*Candidatus* Ruthia magnifica is the chemosynthetic gill endosymbiont of the giant clam, *Calyptogena magnifica* Boss and Turner (Bivalvia: Vesicomyidae) (Figure 1). Vesicomyid clams are conspicuous fauna at many deep-sea hydrothermal-vent and cold-seep habitats. *Candidatus* R. magnifica, a member of the phylum *Gammaproteobacteria*, falls within the vesicomyid symbiont clade which is a sister group to vent and seep mussel chemosynthetic symbionts of the subfamily *Bathymolidinae* (Figure 2).

## Project history

The *Calyptogena magnifica* symbiont *Candidatus* Ruthia magnifica was selected for sequencing because this symbiosis is one of the dominant macrofauna at vent sites in the eastern Pacific Ocean. Knowledge of the metabolic capabilities of this symbiosis provides new perspectives on the coupling of carbon and sulfur fluxes in the deep-sea, a substantial reservoir in the global carbon cycle. In addition, this genome provides insights into the origin and evolution of autotrophic endosymbiosis. This project was funded by a US Department of Energy as part of the Joint Genome Institute Community Sequencing Program.

The complete genome sequence was finished in January 2006 and originally described in Newton et al. 2007 [9]. The GenBank accession number for the symbiont genome is CP000488.1 and is listed in the Genomes OnLine Database (GOLD) as project Gc00468. A summary of the project information is shown in Table 2.

**Table 2:** Project information

**MIGS ID**	**Property**	**Term**
MIGS-31	Finishing quality	Finished
MIGS-28	Libraries used	3kb pUC, 8kb pMCL, and fosmid
MIGS-29	Sequencing platforms	Sanger: ABI3730
MIGS-31.2	Fold coverage	~14×
MIGS-30	Assemblers	Parallel phrap
MIGS-32	Gene calling method	Glimmer
	Sequencing Center	DOE Joint Genome Institute
	Funding Agency	DOE
	Genome Database release	March 1, 2007
	Genbank ID	CP000488.1
MIGS-1.1	NCBI project ID	16841
	Genbank Date of Release	November 29, 2006
	GOLD ID	Gc00468
	Project relevance	Vent ecosystems, Chemosynthetic symbiosis, Environmental microbiology

## Specimen collection and DNA extraction

*Calyptogena magnifica* clams were collected using DSV Alvin at the East Pacific Rise, 9°N vent field, during a cruise on the R/V Atlantis in December 2004. Symbiont containing gills were dissected out of the clams, frozen in liquid nitrogen, and kept at -80°C until processed in the lab. Gill tissues were ground in liquid nitrogen, placed in lysis buffer (20 mM EDTA, 10 mM Tris-HCl, pH 7.9, 0.5 mg/ml lysozyme, 1% Triton X-100, 200 mM NaCl, 500 mM guanidine-HCl,) and incubated at 40ºC for 2 hr. After subsequent RNase (20 μg/ml, 37°C, 30 min) and proteinase K (20 μg/ml, 50ºC, 1.5 hr) treatments, the samples were centrifuged and the supernatant was transferred onto Qiagen Genomic Tip columns and processed according to manufacturer’s protocol (QIAGEN, Valencia, CA).

## Genome sequencing and assembly

The genome was sequenced by Sanger sequencing of 3kb, 8kb and fosmid libraries. All general aspects of construction and sequencing performed at the JGI can be found on the JGI website (http://www.doe-jgi.gov).

Briefly, 22.15 Mb of phred Q20 sequence were generated: 9.43 Mb from 13,755 reads from the small insert pUC library, 8.79 Mb from 13,824 reads from the medium insert pMCL library, and 3.93 Mb from 9,216 reads from the fosmid library. The DNA sequences derived from the *Candidatus* Ruthia magnifica libraries were estimated to be 20% contaminated with the *Calyptogena magnifica* host genome. Although this level of contamination could confound finishing efforts, the bacterial genome was readily identifiable in this study. The 36,795 sequencing reads were blasted against a database containing all mollusk sequences available in Genbank and the 4× draft gastropod *Lottia gigantea* genome sequence available at the JGI. A total of 498 reads were removed based on hits to this mollusk database.

The remaining 24,595 reads were base called, vector trimmed, and assembled using parallel phrap. One large, bacterial scaffold containing the *Candidatus* R. magnifica 16S rRNA gene resulted. The R. magnifica scaffold consisted of only 2 contigs spanned by 33 fosmid clones, contained 17,307 reads, 1,156,121 consensus bp, was covered by an average read depth of 14×, and had a G+C content of 34%. The next largest scaffold was only 29 kb long, with an average read depth of ~7× and an average G+C content of 55%. BLASTn indicated that this latter scaffold encoded ribosomal genes closely related to those of *Caenorhabditis briggsae* and its binning (based on GC content and read depth) with a small scaffold containing the *C. magnifica* 18S rRNA gene confirmed its eukaryotic host origin.

## Genome annotation

The DNA sequence was submitted to the TIGR auto-annotation pipeline (currently hosted at JCVI). Included in the pipeline is gene finding with Glimmer [14], Blast-extend-repraze (BER) searches, HMM searches, TMHMM searches, SignalP predictions, and automatic annotations from AutoAnnotate. The output from the TIGR Annotation Service was transferred to a MySQL database. Additional gene prediction analysis and manual functional annotation was performed using Manatee (http://manatee.sourceforge.net) [9].

## Metabolic network analysis

The metabolic Pathway/Genome Database (PGDB) was computationally generated by the Pathologic program using Pathway Tools software version 14.0 [15] and MetaCyc version 13.1 [16], based on annotated EC numbers and a customized enzyme name mapping file. The PGDB has not been subjected to manual curation and may contain errors.

## Genome properties

**Fig. 3:**
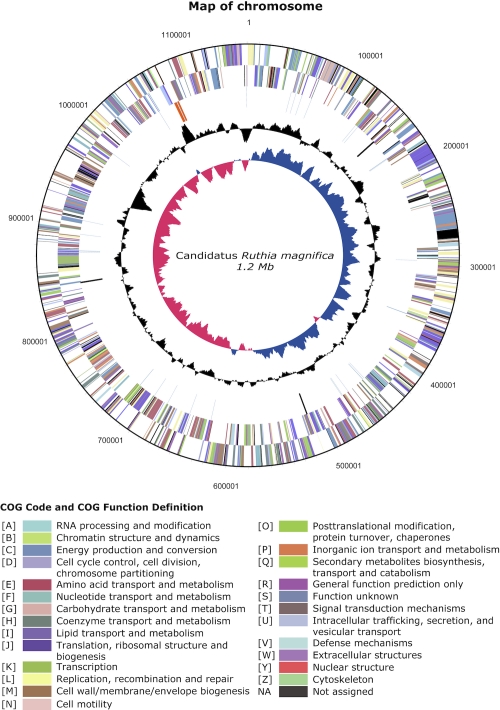
A circular representation of the *Candidatus* R. magnifica genome. The innermost and second circle highlight GC skew and GC content (%) respectively. The third circle shows RNA genes (tRNAs blue, rRNAs orange, other RNAs black). The fourth and fifth circles show the distribution of genes on the reverse and forward strand respectively (colored by COG categories).

**Figure 4:**
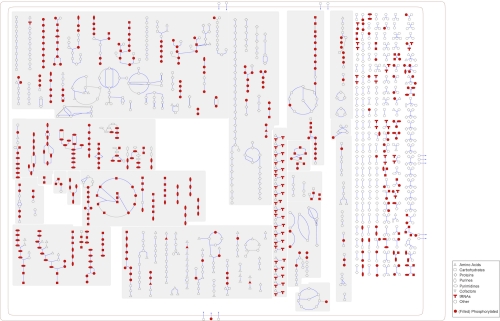
Schematic cellular overview of all pathways of *Candidatus* R. magnifica generated using Pathway Tools software version 14.0 [15]. Nodes represent metabolites, with shapes indicating classes of metabolites. Lines represent reactions.

The genome consists of one circular chromosome with 1,160,782 bp (Figure 3). For the complete genome, 1,118 genes were predicted, 1076 of which are protein-coding genes. 837 of the protein coding genes were assigned to a putative function with the remaining annotated as hypothetical proteins. The properties and the statistics of the genome are summarized in Table 3. The distribution of genes into COG functional categories is presented in Table 4. A cellular overview diagram is presented in Figure 4, followed by a summary of metabolic network statistics shown in Table 5.

**Table 3:** Nucleotide content and gene count levels of the genome

**Attribute**	**Value**	**Total^a^**
Genome size (bp)	1160782	100
DNA G+C content (bp)	395054	34.0
DNA coding region (bp)	976503	84.1
Total genes^b^	1118	100
RNA genes	42	3.8
rRNA genes	3	0.3
tRNA genes	36	3.2
Other RNA genes	3	0.3
Protein-coding genes	1076	96.2
Protein coding genes with function prediction	837	74.9
Genes in paralog clusters	243	21.7
Protein coding genes connected to KEGG pathways	496	44.4
Genes assigned to COGs	932	83.4
Genes with signal peptides	131	11.7
Genes with transmembrane helices	224	20.0
CRISPR repeats	0	0

a) The total is based on either the size of the genome in base pairs or the total number of protein coding genes in the annotated genome.

**Table 4:** Number of genes associated with the general COG functional categories

**Code**	**Value**	**%age**	**Description**
J	137	13.50	Translation, ribosomal structure and biogenesis
A	1	0.10	RNA processing and modification
K	32	3.15	Transcription
L	61	6.01	Replication, recombination and repair
B	0	0.0	Chromatin structure and dynamics
D	13	1.28	Cell cycle control, mitosis and meiosis
Y	0	0.0	Nuclear structure
V	9	0.89	Defense mechanisms
T	17	1.67	Signal transduction mechanisms
M	73	7.19	Cell wall/membrane biogenesis
N	0	0.0	Cell motility
Z	0	0.0	Cytoskeleton
W	0	0.0	Extracellular structures
U	24	2.36	Intracellular trafficking and secretion
O	74	7.29	Posttranslational modification, protein turnover, chaperones
C	90	8.87	Energy production and conversion
G	31	3.05	Carbohydrate transport and metabolism
E	99	9.75	Amino acid transport and metabolism
F	39	3.84	Nucleotide transport and metabolism
H	94	9.26	Coenzyme transport and metabolism
I	36	3.55	Lipid transport and metabolism
P	45	4.43	Inorganic ion transport and metabolism
Q	11	1.08	Secondary metabolites biosynthesis, transport and catabolism
R	77	7.59	General function prediction only
S	52	5.12	Functions unknown
-	186	16.64	Not in COGs

a) The total is based on the total number of protein coding genes in the annotated genome.

**Table 5:** Metabolic Network Statistics

**Attribute**	**Value**
Total genes	1117
Enzymes	430
Enzymatic reactions	702
Pathways	115
Metabolites	565

## Insights from the genome sequence

The *Candidatus* R. magnifica genome has revealed striking differences between the chemosynthetic endosymbiont genomes and those of other obligate mutualistic symbionts for which genomic data are available. The genome is small (1.1 Mb) and has a low G+C content (34%) compared to free-living sulfur oxidizing proteobacteria [9]. These common features of endosymbionts are likely the result of genome reduction and accumulation of point mutations that occur over evolutionary time across diverse symbiont species [17]. This trend has been observed in recently evolved symbioses such the insect endosymbionts (30-250 Ma) [18], as well as in chloroplasts (~1,800-2,100 Ma) [19].

However, *Candidatus* R. magnifica stands out in that its genome is relatively large for a maternally transmitted endosymbiont. For example, the genomes of the Gammaproteobacterial *Buchnera* which are endosymbionts of aphids, are ~85% smaller than closely related free-living species like *E. coli.* In contrast, the genome of *Candidatus* R. magnifica is ~24% the size of *E. coli K12* and ~55% smaller than *Thiomicrospira crunogena,* a free-living, Gammaproteobacterial, sulfur-oxidizing chemoautotroph isolated from vents [20].

The genome lacks any form of mobile DNA content. Neither transposon- nor phage-related sequences were identified except for the putative prophage repressor gene LexA (EC 3.4.21.88).

The genome encodes enzymes specific for carbon fixation via the Calvin cycle; including a form II ribulose 1,5-bisphosphate carboxylase-oxygenase (RuBisCO, EC 4.1.1.39) and phosphoribulokinase (EC 2.7.1.19) [9]. Energy for carbon fixation appears to be derived from sulfur oxidation via the “sulfur oxidation (sox) pathway” and dsr (dissimilatory sulfite reductase) pathway [9].

Remarkably, the genome lacks the Calvin cycle homologs sedoheptulose 1,7-bis-phosphatase (SBPase, EC 3.1.3.37) and fructose 1,6-bis-phosphatase (FBPase, EC 3.1.3.11), suggesting that the regeneration of ribulose 1,5-bisphosphate may not follow conventional pathways [9]. Instead, the genome contains a reversible pyrophosphate-dependent phosphofructokinase (EC 2.7.1.90) homolog that may be used to generate fructose 6-phosphate [21].

The central intermediary metabolism of *Candidatus* R. magnifica produces all the intermediates necessary for the synthesis of amino acids, nucleotides, fatty acids, vitamins and cofactors, which are thought to be supplied to the host [9]. Notably, the symbiont lacks homologs of fumarate reductase, succinate dehydrogenase, and succinyl-coA synthase. However, the genome encodes isocitrate lyase, part of the glyoxylate shunt, suggesting succinate production from isocitrate [22].

Although able to synthesize 10 vitamins/cofactors, the cobalamin (B12) biosynthesis pathway is conspicuously absent [9]. Since cobalamin is a cofactor for methionine synthase [23] and since *Candidatus* R. magnifica encodes a cobalamin-independent methionine synthase, the host might not require cobalamin.

Several transporters involved in chemoautotrophy (sulfate exporters), nitrogen assimilation (nitrate and ammonium transporters), inorganic compounds (TrkAH, MgtE family, CaCA family and PiT family), and heavy metals (ZnuABC, RND superfamily, iron permeases) were identified [9].

The diverse metabolic capabilities of *Candidatus* R. magnifica*,* inferred from the genome sequence, confirm and extend our understanding of host nutritional dependency.
